# The Rolla District Medical Society

**Published:** 1880-07

**Authors:** 


### THE ROLLA DISTRICT MEDICAL SOCIETY.

Dr. J. E. Thompson called the attention of the Society to the hypodermic use of ergot in hemorrhage, and reported a case of hemorrhage from the ileum in typhoid fever arrested promptly by ergot subcutaneously. Squibb's fluid extract was used undiluted in half drachm doses injected into the arm and repeated every thirty minutes until three doses were given. In a very few minutes after the last dose the bleeding ceased and did not recur. Five minim doses were given by the mouth every three hours for twenty-four hours. The patient, a young lady of fifteen years, recovered—convalescence having been established at the end of the fourth week. Quite an animated discussion followed between Drs. Gibson, Fetzer, Lenox, Coffee and Thompson.

Dr. Thompson stated that his attention had been called to the subcutaneous use of ergot in hemorrhages by Prof. R. M. King, of St. Louis. In the case cited the bleeding surface could not have been reached in any other way, as the stomach rejected everything ingested.

Prof. Bauer addressed the Society at length upon fracture of the shaft of the femur. He called attention to the method of treating such cases advocated by Dr. Hamilton, New York City, taking strong and logical grounds against Dr. Hamilton's assertion that "shortening must take place from contraction of the muscles." He strongly recommended the double-inclined plane as the only rational appliance, and showed conclusively by demonstrations the utter fallacy of attempting to prevent muscular contraction by weights attached to straight or extension splints.

Dr. Craig presented a case of supposed incomplete fracture of the tibia. Upon careful examination, however, the case was pronounced to be one of traumatic periostitis involving more than half of the shaft of the tibia and extending to the fibula. Professor Bauer stated at length and in a very explicit manner the pathology of periostitis and osteomyelitis, and proposed the subcutaneous division of the periosteum as a means of instant relief from the excessive tenderness and pain. He accordingly split up the periosteum, when all tenderness on pressure subsided. A flannel bandage was applied and the man went away greatly relieved.

Dr. J. E. Thompson presented a boy six years old presenting antero-posterior curvature of the thoracic vertebra, of two years' standing. From the history of the case he concluded that it was of traumatic origin. Sayre's plaster jacket had been tried, but with no benefit. The flexibility of three or four of the dorsal vertebræ had been destroyed. Dr. Bauer advised, as in other joint diseases, rest in the recumbent position, and immobilization. These had brought more relief than all the anti-strumous remedies the world had ever produced.

SECOND DAY, May 27th.—Society was called to order by the President at 8 o'clock A.M.

Dr. Thompson, chairman of the Memorial Committee, reported resolutions in memorium of William E. Glenn, M.D., formerly of Rolla, who died at Leadville, Colorado, last January. These were ordered to be spread upon the journal of the Society.

Dr. S. F. Arthur, of Edgar's Springs, reported a case quite extraordinary in many of its features. A *post-mortem* examination was denied, hence the diagnosis was wrapped in doubt. Most of the members who expressed themselves were of opinion that it was a case of phlebitis Professor Bauer spoke of the importance of *post-mortem* examinations and the means physicians should resort to in order to obtain them. He spoke of the dangers of thrombus and embolism as a result of phlebitis.

Dr. Coffee reported a case of calcarious degeneration of the arteries of the brain. Dr. S. C. Gibson said he had seen the case referred to by Dr. Coffee frequently, and regarded it as one of general atheromatous degeneration. The man was over sixty years of age. Over two years ago he began to complain of urinismus. Shortly the arteries on the forehead became plainly visible and very tortuous. Every artery accessible felt like a wet quill. He had hemiplegia of the right side. The sensitive nerves were benumbed; you may prick the skin and it will be thirty or forty seconds before any sensation will be produced; his memory is defective, accompanied with aphasia; he cannot say what he wants to say, but says Jim for John. He is subject to "falling fits." Professor Bauer said the man evidently had progressive softening of the brain, and when a large clot forms he will die.

Dr. Gibson reported a case of gun-shot wound of the scalp which produced a clot resulting in monoplegia.

Dr. Thompson presented a man with hydrarthrosis of the left knee joint, the result of subacute synovitis. It had been six months since he first noticed that his knee was swollen and stiffened. It had not been painful at any time. About five weeks ago he had applied a plaster bandage, and since that time had kept the joint immobilized in the straight position, and, by the application of ninety per cent. tincture of iodine he had hoped to get rid of the flnid by absorbtion; but it was evident that the joint would have to be opened, and asked Prof. Bauer to perform the operation before the Society. Dr. Bauer bandaged the leg from the foot to above the joint with flannel so as to force the fluid into the cul-de-sac above the patella, then plunged a trocar into it, when three or four ounces of strongly alkaline serum came out with great force. The joint was then firmly bandaged, which was to remain for twenty-four to thirty-six hours, when it might be removed. Dr. Bauer dwelt at considerable length upon the pathology of joint diseases and their treatment.

Dr. Coffee read a lengthy paper on "Labor." Dr. J. E. Thompson reported a case of puerperal eclampsia which ended in recovery.

